# Cerebrospinal Fluid Concentrations of the Synaptic Marker Neurogranin in Neuro-HIV and Other Neurological Disorders

**DOI:** 10.1007/s11904-019-00420-1

**Published:** 2019-01-16

**Authors:** Aylin Yilmaz, Dietmar Fuchs, Richard W. Price, Serena Spudich, Kaj Blennow, Henrik Zetterberg, Magnus Gisslén

**Affiliations:** 1 Department of Infectious Diseases, Institute of Biomedicine, Sahlgrenska Academy, University of Gothenburg, 416 45 Gothenburg, Sweden; 2 Division of Biological Chemistry, Biocenter, Innsbruck Medical University, Innsbruck, Austria; 3 Department of Neurology, University of California San Francisco, San Francisco, CA USA; 4 Department of Neurology, Yale University, New Haven, CT USA; 5 Institute of Neuroscience and Physiology, Department of Psychiatry and Neurochemistry, University of Gothenburg, Gothenburg, Sweden; 6 Clinical Neurochemistry Laboratory, Sahlgrenska University Hospital, Molndal, Sweden; 7 Department of Neurodegenerative Disease, UCL Institute of Neurology, Queen Square, London, UK; 8 UK Dementia Research Institute at UCL, London, UK

**Keywords:** HIV, Cerebrospinal fluid, Neurogranin

## Abstract

**Purpose of Review:**

The aim of this study was to examine the synaptic biomarker neurogranin in cerebrospinal fluid (CSF) in different stages of HIV infection and in relation to what is known about CSF neurogranin in other neurodegenerative diseases.

**Recent Findings:**

CSF concentrations of neurogranin are increased in Alzheimer’s disease, but not in other neurodegenerative disorder such as Parkinson’s disease, frontotemporal dementia, and Lewy body dementia. Adults with HIV-associated dementia have been found to have decreased levels of neurogranin in the frontal cortex, which at least to some extent, may be mediated by the proinflammatory cytokines IL-1β and IL-8.

**Summary:**

CSF neurogranin concentrations were in the same range for all groups of HIV-infected individuals and uninfected controls. This either indicates that synaptic injury is not an important part of HIV neuropathogenesis or that CSF neurogranin is not sensitive to the type of synaptic impairment present in HIV-associated neurocognitive disorders.

## Introduction

Neurogranin is a neuron-specific post-synaptic protein, abundant in excitatory neurons in the cortex, hippocampus, and amygdala [1]. It plays an important part in synaptic plasticity, enhancing synaptic strength by regulating the availability of calmodulin [2, 3]. This process is an essential part in long-term potentiation (LTP), a process believed to be essential to generate memories. Neurogranin knock-out mice display loss of spatial and emotional learning as well as a decrease in LTP induction resulting in disorientation [4]. In humans, synaptic dysfunction (assessed via CSF neurogranin) has been associated with memory performance [5].

Neurogranin can be quantified in CSF with enzyme-linked immunosorbent assay (ELISA), and it has been studied in various neurocognitive disorders. Alzheimer’s disease affects the brain regions where neurogranin is mainly expressed, i.e., the hippocampus, amygdala, and the neocortex. Synapse loss has been shown to be an early event, occurring prior to neuronal death and cognitive decline [6–8]. Neurogranin levels are markedly reduced in the frontal cortex and hippocampus in Alzheimer’s disease, indicating loss of post-synaptic elements [9]. The development of novel anti-neurogranin monoclonal antibodies has made it possible to quantify low levels of neurogranin in CSF [10•]. Several studies have shown that CSF neurogranin concentrations are elevated in patients with mild cognitive impairment due to Alzheimer’s disease as well as in patients with Alzheimer’s disease [10•, 11]. There is a correlation between levels of CSF neurogranin and the severity of cognitive decline and brain atrophy in early stages of the disease [12, 13].

Since synapse degeneration occurs in all neurodegenerative processes, it is an unexpected finding that CSF neurogranin concentrations are not increased in other neurodegenerative disorders such as Parkinson’s disease, frontotemporal dementia, Lewy body dementia, progressive supranuclear palsy, or multiple system atrophy, indicating that high CSF neurogranin might be specific for Alzheimer’s disease [14, 15•]. This could be explained by the fact that the main brain regions affected by Alzheimer’s disease are also the regions with the highest expression of neurogranin.

In regard to multiple sclerosis and neuroinflammatory conditions, there is very little published on CSF neurogranin. Although axonal damage may be pronounced in relapsing-remitting multiple sclerosis, in one published study, there were no signs of dendritic spine involvement and CSF neurogranin levels were normal [16].

Soon after transmission, HIV can be detected in the cerebrospinal fluid (CSF) in most individuals [17, 18]. If left untreated, it will lead to a neurodegenerative process with inflammation and neuronal loss that eventually can manifest with HIV encephalitis presenting as subacute HIV-associated dementia (HAD) [19, 20]. Since neurons are not infected by HIV, other mechanisms must be involved in the neuropathological damage. In addition to neuronal injury and death, synaptic disruption also contributes to neurocognitive impairment in HIV-infected individuals. The pathogenesis of neurodegeneration, including synaptic injury, is still only partially understood, but chronic immune activation and a combination of secondary effects due to both viral and host factors have been suggested [21–23].

Several CSF biomarkers reflect the intrathecal immune activation and neuronal damage in HIV infection. Neopterin is an important marker of cell-mediated immune activation. It is mainly produced by activated macrophages/monocytes, and its CSF levels increase with increasing immunosuppression in untreated HIV-infected individuals and are highest in patients with HAD and CNS opportunistic infections [24, 25]. CSF neurofilament light protein (NFL) is a sensitive marker of HIV-induced axonal injury [25, 26, 27•]. CSF NFL concentrations are highest in individuals with HAD where neuronal damage and loss are prominent, but some untreated individuals without neurocognitive symptoms with low CD4^+^ T cell counts may also have high levels.

HIV and Alzheimer’s disease share some similar features in neuropathology. One example is the increased deposition of hyperphosphorylated tau in the hippocampus [28], but there are also differences. Green et al. found ß-amyloid plaques in brains from HIV-infected individuals [29], whereas others [28, 30] have not detected any difference when compared to HIV-negative age-matched controls.

There are also similarities as well as differences in CSF biomarkers between HAD and Alzheimer’s disease. Typical changes in CSF biomarkers in Alzheimer’s disease include increases in total tau (t-tau) and phosphorylated tau (p-tau) and a decrease in Aβ_1–42_ [31]. In HIV-infected individuals with neuronal injury and cognitive impairment, t-tau can be elevated but p-tau is generally not [32–34].

There is a potential for synaptic injury in HIV infection by exposure to viral proteins such as Tat and gp120 and proinflammatory substances released by activated cells in the CNS [22]. There is, however, only scarce information about the role or perturbation of neurogranin in HIV infection, and to our knowledge, nothing about CSF neurogranin levels. In a recently published study, it was found that patients with HIV-associated neurocognitive disorders had significantly reduced expression of neurogranin in frontal cortex tissues compared to uninfected controls [35].

HIV-infected individuals now have long lifespans and therefore are at risk of being affected by diseases that come with age, such as various neurocognitive disorders. It is important to be able to correctly diagnose these conditions. CSF biomarkers add valuable information in situations like this, since they can aid in discriminating between different conditions.

To test the hypothesis that synapses are involved in the neuropathogenic pathway in HIV-1 infection, we have analyzed CSF neurogranin in a cohort of HIV-infected individuals thoroughly classified by systemic progression, CNS symptomatic presentation, and antiretroviral therapy (ART).

### Methods

In this cross-sectional study, we used archived CSF samples from two academic centers: Sahlgrenska University Hospital, Gothenburg, Sweden, and San Francisco General Hospital, University of California San Francisco (UCSF), USA. All research protocols and informed consents were reviewed and approved by an ethics committee.

The study consisted of 138 HIV-infected individuals divided into six groups and 13 HIV-negative controls. HIV-infected participants were divided into four groups with untreated subjects without neurological impairment (neuroasymptomatic; NA) stratified by CD4^+^ T cell count into CD4 < 50 cells/μL (*n* = 25), CD4 50–199 cells/μL (*n* = 25), CD4 200–349 cells/μL (*n* = 25), and CD4 > 350 cells/μL (*n* = 30), subjects with HAD (*n* = 11), and subjects on suppressive ART without signs of neurological impairment (*n* = 33).

### CSF Samples and Analytical Methods

Cell counts, HIV RNA, and proteins were analyzed immediately after sampling and remaining aliquots were centrifuged and frozen at − 70 °C until further analysis.

CSF neurogranin was measured by an in-house ELISA using Ng7 as capturing antibody and polyclonal Ng anti-rabbit antibody (ab23570; Upstate Biotechnology, Lake Placid, NY, USA) as detecting antibody, as previously described in detail [11]. All samples were analyzed in one round of experiments using one batch of reagents by board-certified laboratory technicians who were blinded to clinical data. The lower limit of quantification was 125 pg/mL, and intra-assay coefficients of variation were below 10%. CSF NFL was measured using a commercially available sandwich ELISA (NF-light® ELISA kit, UmanDiagnostics AB, Umeå, Sweden). Upper reference values are age-dependent [27•]. CSF neopterin was analyzed using a commercially available immunoassay (BRAHMS, Berlin, Germany) with an upper normal reference value of 5.8 nmol/L [24].

HIV RNA in CSF and plasma was measured using the Roche Amplicor Monitor version 1.5, Roche Taqman assay version 1 or 2 (Hoffman La-Roche, Basel, Switzerland), or Abbott RealTime HIV-1 assay (Abbott Laboratories; Abbott Park, Illinois, USA). Lower limit of detection was 20–40 copies/mL.

### Statistical Methods

Statistical analysis was made with SPSS version 21 (IBM for Mac) and graphs made by GraphPad, Prism 7.0. Correlations were analyzed by Pearson correlation analysis. One-way ANOVA with Tukey’s multiple comparisons tests were used for adjusted *p* values for differences between groups. To reduce skewness, the following parameters were log transformed: plasma HIV RNA, CSF HIV RNA, CSF NFL, CSF neopterin, CSF neurogranin, and CD4^+^ T cell count.

## Results

**Fig. 1:**
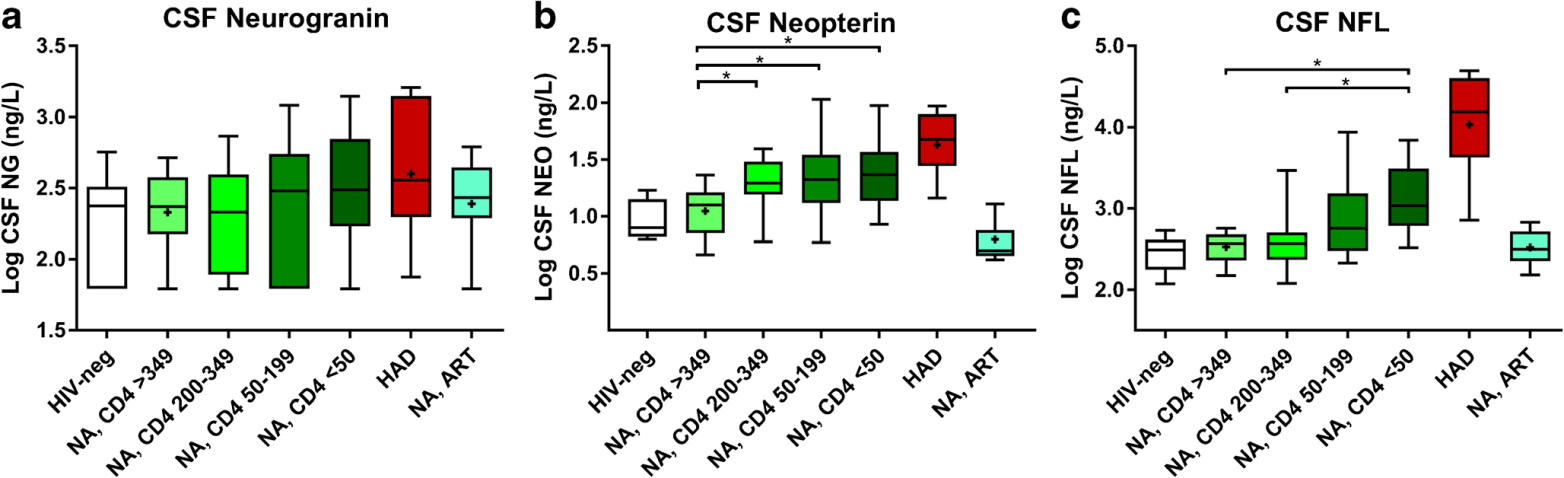
Log concentrations of (**a**) cerebrospinal fluid (CSF) neurogranin, (**b**) CSF neopterin, and (**c**) CSF NFL for the seven groups of participants: HIV-negative; NA neuroasymptomatic untreated individuals, subgrouped by level of blood CD4^+^ T cell count to CD4 < 50, 50–199, 200–349, and > 349 T-cells/μL; individuals with diagnosed HAD (HIV-associated dementia); and individuals on suppressive antiretroviral therapy without signs of neurological impairment

**Fig. 2:**
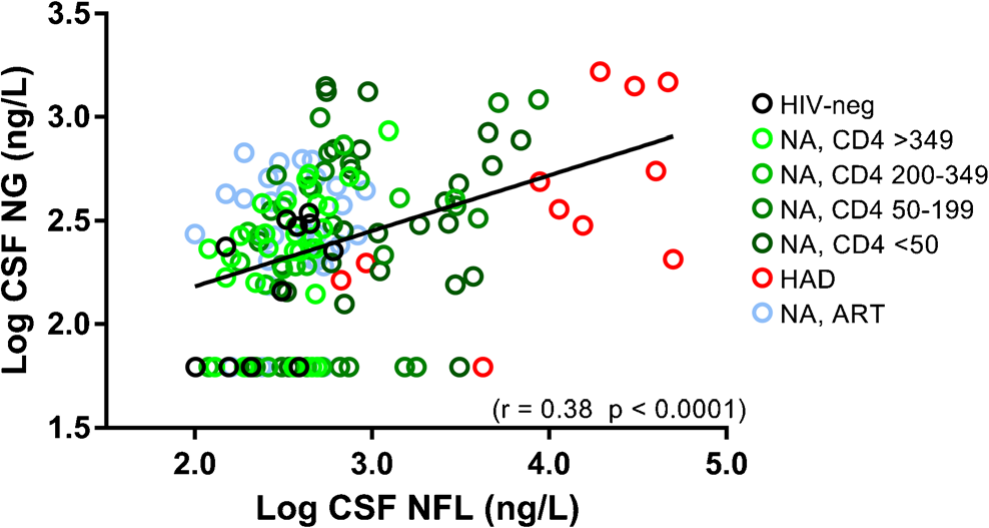
Correlation between cerebrospinal fluid (CSF) NFL and CSF neurogranin with the same color-coding for the seven different groups of patients as in Fig. 1

Patient characteristics are presented in Table 1. In contrast to CSF NFL, there were no significant differences in CSF neurogranin levels between any of the studied groups; although, there was a relatively wide range within each group (Fig. 1a). CSF neurogranin concentrations were in the same range for HIV-negative controls as all groups of HIV-infected individuals. CSF NFL concentrations were highest in the HAD group and significantly higher CSF NFL was also found in neuroasymptomatic individuals with low CD4^+^ T cell counts as compared to those with higher CD4^+^ T cell counts and HIV-negative controls. Participants with HAD and neuroasymtpomatic individuals with low CD4^+^ counts also had higher CSF neopterin and NFL levels compared with the other groups (Fig. 1b, c). A weak but statistically significant correlation was found between CSF neurogranin and CSF NFL concentrations (*r* = 0.38, *p* < 0.0001) (Fig. 2). The correlation between CSF neurogranin and CSF neopterin was even weaker, but still significant (*r* = 0.18, *p* < 0.05), but stronger between CSF NFL and CSF neopterin (*r* = 0.47, *p* < 0.0001).

**Table 1:** Patient characteristics

	N	Age (years)	Gender (female)	CD4 (cells/μL)	Plasma HIV RNA
Group		Median (IQR)	Number (%)	Median (IQR)	Log_10_ copies/mL
HIV negative	13	39 (29–47)	3 (23)	702*	N.A
HIV neuroasymptomatic					
CD4 > 350	30	41 (32–45)	10 (33)	462 (397–618)	4.17 (3.72–4.57)
CD4 200–349	25	39 (32–48)	9 (36)	240 (220–290	4.74 (4.32–5.04)
CD4 50–199	25	42 (34–51)	13 (52)	110 (90–139)	5.09 (4.66–5.42)
CD4 < 50	25	44 (36–48)	9 (36)	20 (10–30)	5.49 (5.00–5.90)
HIV-associated dementia	11	43 (40–57)	0 (0)	60 (39–137)	5.69 (5.46–5.82)
Neuroasymptomatic, ART	33	44 (36–54)	13 (39)	580 (450–830)	< 1.30 (< 1.30–< 1.30)

*N* number, *IQR* interquartile range, *NA* not analyzed, *ART* antiretroviral therapy, *CSF* cerebrospinal fluid

*CD4 only available for four controls, IQR not presented

## Discussion

The pathogenesis leading to neuronal injury and death in HIV-infection is complex and far from completely understood. Our aim was to investigate if early synaptic dysfunction could precede axonal injury in HIV-infected individuals, but CSF neurogranin concentrations were in the same range for all participants, uninfected controls, individuals with CD4+ T cell counts ranging from less than 50 to more than 349, individuals on suppressive ART, and even individuals with HAD. This either indicates that synaptic injury is not an important part of HIV pathogenesis in the CNS or, which probably is more likely, that CSF neurogranin is not a sensitive marker for the type of synaptic impairment that may be ongoing in HIV-associated neurocognitive disorders, in analogy with several other neurodegenerative disorders [15•].

A study published earlier this year found that individuals with HAD had significantly lower expression of neurogranin in tissue from frontal cortex [35]. CSF neurogranin concentrations were not evaluated. The number of participants with HAD was, however, very low (*n* = 3), and even though they were on ART, they had high plasma viral loads, so the results need to be interpreted with caution.

In Alzheimer’s disease, there is also a marked reduction of neurogranin levels in frontal cortex and hippocampus, the same areas of the brain where neurogranin is mostly expressed. CSF neurogranin levels are increased in Alzheimer’s disease, but not in other studied neurodegenerative disorders [15•]. When quantified with the same method as in this study, individuals with Alzheimer’s disease had significantly higher CSF neurogranin levels than the other groups (median and interquartile range), 463 (275–669) pg/mL in Alzheimer’s disease, compared with 196 (120–297) pg/mL in healthy controls, 120 (120–304) pg/mL in Lewy body dementia, 156 (120–283) pg/mL in Parkinson’s disease, 188 (120–302) pg/mL) in progressive supranuclear palsy, and 191 (120–265) in multiple system atrophy. This could perhaps be due to the fact that different brain regions are affected by various disorders or that the degree of synaptic injury, and hence, neurogranin levels in CSF vary. It has therefore been suggested that CSF neurogranin could be used as a specific biomarker for Alzheimer’s disease.

With an aging population of HIV-infected individuals on ART, it is important to be able to discriminate neurological impairment caused by HIV itself from other neurocognitive disorders. CSF biomarkers are of great value in situations like this, since they can reflect different aspects of the pathogenesis of HIV CNS infection. CSF neurogranin could, perhaps, in combination with other CSF biomarkers, contributes to differentiating between HAD and other causes for neurocognitive decline, for example Alzheimer’s disease [36].

Since HIV does not infect neurons, other mechanisms are responsible for the synaptodendritic injury, neuronal dysfunction, and apoptosis that occurs. Although the exact pathogenesis remains unclear, some of the key steps in this process involve viral invasion of the CNS, which triggers local immune activation mediated by infected perivascular macrophages and microglia interacting with astrocytes. CSF neopterin is mainly produced by macrophages and related cells and is one of the most used markers of cell-mediated intrathecal immune activation [37, 38]. We found the same pattern for CSF neopterin levels as in several other studies [24, 39], with the highest values in those with HAD and thereafter in those with low CD4^+^ T cell counts. Increased CSF NFL levels are common in advanced stages of HIV-infection, particularly in individuals with HAD [24, 40], something we also found in this study.

This study has several limitations. It has a retrospective cross-sectional design and even though we did not find any significant differences in CSF neurogranin levels in the various groups of HIV-infected participants, it could be of interest to follow CSF neurogranin levels in a longitudinal study, especially after initiation of ART. The total number of participants was relatively large, but some of the individual groups were limited in size.

## Conclusions

CSF concentrations of neurogranin are increased in Alzheimer’s disease, but not in other neurodegenerative disorder such as Parkinson’s disease, frontotemporal dementia, Lewy body dementia, progressive supranuclear palsy, or multiple system atrophy. Our results show that CSF neurogranin levels are not increased during any stage of HIV-infection compared to uninfected controls. Since neurogranin is a biomarker of synaptic injury, these findings either indicate that axonal injury occurs in HIV-infected individuals without preceding synaptic damage or that CSF neurogranin is not sensitive to the type of synaptic impairment that may be present in HIV-associated neurocognitive disorders.
